# Case mix and outcomes for admissions to UK adult, general critical care units with chronic obstructive pulmonary disease: a secondary analysis of the ICNARC Case Mix Programme Database

**DOI:** 10.1186/cc3719

**Published:** 2005-06-17

**Authors:** Martin J Wildman, David A Harrison, Anthony R Brady, Kathy Rowan

**Affiliations:** 1 Lecturer in Health Services Research, London School Hygiene and Tropical Medicine, London, UK; 2 Statistician, Intensive Care National Audit and Research Centre (ICNARC), London, UK; 3 Senior Statistician, ICNARC, London, UK; 4 Director, ICNARC, London, UK

## Abstract

**Introduction:**

Chronic obstructive pulmonary disease (COPD) is a common cause of admission to intensive care units (ICUs) in the UK. This report describes the case mix and outcomes of these patients and explores associations of measures of case mix available in the first 24 hours with outcome.

**Method:**

We conducted a secondary analysis of a high quality clinical database, the ICNARC Case Mix Programme Database, of 129,647 admissions to 128 adult, general critical care units across England, Wales and Northern Ireland for the period from 1995 to 2001.

**Results:**

Nonsurgical admissions with COPD accounted for 3752 admissions (2.9% of all admissions). Patients were acidotic (median pH 7.26, interquartile range [IQR] 7.18–7.33), hypercapnic (median arterial CO_2 _tension 8.7, IQR 6.9–10.7) and hypoxic (median arterial O_2 _tension/fractional inspired oxygen gradient 22.9, IQR 17.2–29.6). Overall, 2775 (73.9%) were definitely intubated and 278 (7.4%) were probably intubated in the first 24 hours in the ICU. The median (IQR) ICU length of stay was 4.0 (1.6–9.4) days and the hospital length of stay was 16 (9–29) days. a total of 827 patients (23.1%) died in the admitting ICU and 1322 (38.3%) died during hospital admission. Age, presence of severe respiratory disease, length of stay in hospital before critical care admission, cardiopulmonary resuscitation within 24 hours before admission, intubation status in first 24 hours in critical care, pH, arterial oxygen tension/fractional inspired oxygen gradient, albumin, cardiovascular organ failure, neurological organ failure and renal organ failure all had independent associations with hospital mortality. Respiratory organ failure had a significant independent association with decreased hospital mortality.

**Conclusion:**

Nonsurgical patients with COPD represent an important group of patients admitted to UK ICUs. The presence of single organ respiratory failure in the first 24 hours in critical care identifies patients with a 70% chance of surviving to leave hospital.

## Introduction

The prevalence of chronic obstructive pulmonary disease (COPD) in the UK has been estimated to be 1%, increasing to 5% in men aged 65–74 years and 10% in men older than 75 years [1]. COPD accounted for more than 100,000 hospital admissions in England in 2000/2001 and is the fifth most common cause of mortality in the UK, with 30,000 deaths each year [2]. Exacerbations of COPD may lead to respiratory failure requiring ventilatory support either noninvasively or invasively following endotracheal intubation. A recent UK study [3] suggested that clinicians involved in the emergency care of COPD patients had made a median of 10 intensive care unit (ICU) gatekeeping decisions for COPD patients in the preceding 12 months.

COPD is a progressive disorder that spans a continuum from early mild disease to severe and disabling terminal disease, and prognostic estimates can be important in informing critical care admission decisions [4]. Factors found to have an association with mortality have included those related to diminished functional reserve resulting from the COPD, such as impairments in activities of daily living (ADL) and factors related to the aetiology of respiratory failure [5]; severity of the acute illness, measured using either the acute physiology score of APACHE (Acute Physiology and Chronic Health Evaluation) II [6] or APACHE III [5,7]; and the presence of nonrespiratory organ failures [7,8].

The majority of studies reporting COPD ICU outcomes originate from the USA, and there have only been two recent studies from the UK; one reported on 42 intubated patients in a single centre [9] and another reported hospital mortality of 277 COPD ICU admissions from 24 ICUs in one region of the UK [10].

This report examines the outcomes of COPD patients admitted to ICUs across England, Wales and Northern Ireland, identified using a high quality clinical database. The case mix at ICU admission, outcome and activity associated with these admissions are described. The effect of factors, determined *a priori*, on hospital mortality is investigated.

## Materials and methods

### Case Mix Programme Database

Data were extracted for 129,647 admissions to 128 adult, general critical care units (ICUs including combined intensive care/high dependency units) from the Case Mix Programme Database (CMPD), covering the period from December 1995 to August 2001. Data were collected locally by trained data collectors according to precise rules and definitions, and the data underwent extensive validation before incorporation into the CMPD. The process of data validation and cleaning was described in detail previously [11].

### Selection of data

A standard coding method that classifies information describing the reasons for admission was used to identify the patients, and has been described previously [12]. Surgical patients were identified and excluded when the primary or secondary reason for admission was a surgical code or if the patient was admitted from theatre having undergone all or part of a surgical procedure. Nonsurgical admissions were selected if they had a primary reason for admission of COPD, exacerbation of COPD or emphysema, or any of these three conditions as the secondary reason for admission when pneumonia, right ventricular failure, or left ventricular failure was the primary reason. Patients were assigned to one of three subgroups of COPD (pneumonia in a patient with COPD, right ventricular failure in a patient with COPD, or left ventricular failure in a patient with COPD) if the primary or secondary reason for admission was pneumonia, right ventricular failure, or left ventricular failure, respectively, in a patient in whom the other reason for admission was COPD, exacerbation of COPD, or emphysema. The primary and secondary reasons for admission were the diagnoses made by the clinicians and recorded in the patient notes. As such the diagnoses were not arrived at by applying preset diagnostic criteria but by using the working clinical diagnoses reached during the management of the patients, and will reflect the uncertainty inherent in clinical practice.

In addition, the intubation status of the COPD admissions was categorized on the basis of whether they were definitely intubated within the first 24 hours of admission to the Case Mix Programme (CMP) unit, probably intubated in the first 24 hours, or not intubated in the first 24 hours. The CMPD does not contain a response field solely for the identification of intubation status, but patients with an intubated fractional inspired oxygen (FiO_2_) recorded and those defined as intubated in the field describing the intubation status associated with the lowest arterial oxygen tension (PaO_2_) were identified as definitely intubated. Patients who were probably intubated had a response of 'yes' in the field for mechanical ventilation at admission, or a non-zero value for the lowest or highest ventilated respiratory rate. Patients not intubated in the first 24 hours in the CMP unit fulfilled none of the above criteria.

### Data

Data were extracted on case mix, outcome and activity for nonsurgical COPD admissions to CMP units. Detailed description of the definitions used in the CMPD are described elsewhere [11], but the definitions of particular relevance to the COPD patients are outlined below.

### Case mix

Age at admission and sex were extracted. A history of respiratory functional impairment and comorbidity was recorded using data collected as part of the chronic health evaluation section of the APACHE II score [13]. The respiratory functional impairment has two aspects: 'severe respiratory disease' is defined as patients with permanent shortness of breath with light activity due to pulmonary disease; and 'home ventilation' specifies whether a patient has used or uses home ventilation (excluding continuous positive airway pressure ventilation). Patients with liver disease included those with an episode of hepatic encephalopathy in the previous 6 months or who had evidence of portal hypertension. Patients with cardiovascular comorbidity included patients with fatigue, claudication or angina at rest, in whom any activity increased the symptoms and where the symptoms were ascribed to myocardial or peripheral vascular disease. Patients with chronic renal failure were those requiring chronic renal replacement therapy. Patients with haematological diseases included those with acute myelogenous leukaemia, acute lymphocytic leukaemia, multiple myeloma, chronic myelogenous leukaemia, chronic lymphocytic leukaemia, and lymphoma. Patients with immunosuppression included those with a congenital immunohumoral or cellular immune deficiency state and those receiving chemotherapy defined as the receipt of a drug that can lower resistance to infection and those who have received radiotherapy. AIDS patients were those meeting the current World Health Organization definition. Patients with metastatic cancer had distant metastases documented by surgery, imaging, or biopsy. History of steroid treatment was defined as the receipt of 0.3 mg/kg, or greater, prednisolone (or equivalent) daily for the 6 months before critical care admission. Patients receiving cardiopulmonary resuscitation within 24 hours before unit admission were also identified.

The following physiological variables, selected *a priori*, were extracted from the data collected during the first 24 hours in the CMP unit: lowest pH; arterial carbon dioxide tension from the arterial blood gas with the lowest pH; PaO_2_/FiO_2 _gradient from the arterial blood gas with the lowest PaO_2_; and lowest serum albumin. Organ failures were defined according to Knaus [14] using data describing the patient's status during the first 24 hours in the CMP unit. Acute severity was also measured with the acute physiology scores from APACHE II and APACHE III, and the APACHE II and APACHE III scores.

### Outcome

Survival data were collected at discharge from the CMP unit and at ultimate discharge from an acute hospital.

### Activity

Length of stay in the CMP unit was calculated, as fraction of days, from the date/time of admission to the CMP unit and date/time of discharge from the CMP unit or death in the CMP unit. Total length of stay in hospital was calculated, in days, from the date of original admission to hospital and date of ultimate discharge from hospital or death in hospital. Transfers in from (out to) another critical care unit were identified as admissions whose source of admission to the CMP unit (destination following discharge from the unit) was any ICU or high dependency unit in the same or another hospital. Readmissions to the CMP unit in the same hospital stay were identified from the postcode, date of birth and sex, and confirmed by the participating units.

### Analyses

The overall proportion of admissions to CMP units with nonsurgical COPD was calculated along with the proportion by subgroups: COPD with pneumonia; COPD with right ventricular failure; and COPD with left ventricular failure. Case mix, outcome and activity were described for the overall group and for the subgroups. Admissions of patients younger than 16 years, admissions of patients who stayed for less than 8 hours in critical care, and readmissions within the same hospital stay and transfers from other ICUs were excluded from the calculation of the APACHE II acute physiology score and APACHE II score. Admissions of patients younger than 16 years, admissions of patients who stayed fewer than 4 hours in critical care, and readmissions within the same hospital stay were excluded from the calculation of the APACHE III acute physiology score and APACHE III score.

The effects of case mix factors, specified *a priori*, on ultimate hospital mortality for nonsurgical admissions with COPD were investigated using logistic regression.

Readmissions within the same hospital stay and admissions for whom hospital outcome data were missing were excluded from all analyses relating case mix factors to ultimate hospital mortality. In addition to univariate analyses, the factors were entered into a multiple logistic regression model. Adjusted subgroup effects were calculated for the three specified subgroups from the multiple logistic regression model.

All analyses were performed using Stata 8.0 (StataCorp, College Station, TX, USA).

## Results

### Case mix

Of 129,647 admissions to 128 adult, general critical care units in the CMPD, 3755 (2.9%) of admissions were identified as nonsurgical COPD patients. Table 1 describes measures of case mix for all patients with COPD and the subgroups with pneumonia, right ventricular failure and left ventricular failure. Overall, 35% of patients had severe respiratory disease before hospital admission and 9% had received daily steroids in the 6 months before admission, although only 2% were receiving home ventilation. Comorbidity was relatively rare, with fewer than 1% of patients having any comorbidity other than cardiovascular disease, and with cardiovascular comorbidity present in 2.2% of the group as a whole, 5.9% of those with right ventricular failure and 7.6% of those with left ventricular failure. Patients spent a median of 1 day in hospital before admission to the CMP unit, and 8.3% of patients received CPR in the 24 hours before admission to the CMP unit. Of the group as a whole, 2775 (73.9%) were definitely intubated in the first 24 hours, with an additional 278 (7.4%) probably intubated in the first 24 hours. Patients were acidotic, hypercapnic and hypoxic, and had low serum albumin (Table 1). For the group as a whole the median (interquartile range [IQR]) APACHE II and APACHE III scores were 18 (14–23) and 62 (49–78), respectively. Of all admissions, 12.2% had no organ failures, 49.4% had single organ failure (the majority, 45.2% of all admissions, being respiratory), 27.6% had two organ failures, and had 8.2% three or more organ failures.

**Table 1:** Case mix for admissions with chronic obstructive pulmonary disease by subgroup

	All (*n *= 3752)	I. Pneumonia (*n *= 775)	II. Right VF (*n *= 147)	III. Left VF (*n *= 142)
Age (years; median [IQR])	67.8 (60.5–73.6)	68.7 (61.4–74.0)	68.8 (61.7–74.0)	71.9 (67.2–75.1)
Sex (n [%] male)	1942 (51.8)	401 (51.7)	83 (56.5)	81 (57.0)
				
Past medical history (*n *[%])				
Respiratory impairment				
Severe respiratory disease	1236 (35.0)	232 (31.6)	57 (42.2)	33 (25.2)
Home ventilation	70 (2.0)	6 (0.8)	6 (4.4)	2 (1.5)
Steroid treatment	305 (8.6)	61 (8.3)	7 (5.2)	8 (6.1)
Comorbidity:				
Liver disease	6 (0.2)	3 (0.4)	0 (0.0)	0 (0.0)
Cardiovascular disease	79 (2.2)	11 (1.5)	8 (5.9)	10 (7.6)
Chronic renal failure	11 (0.3)	1 (0.1)	0 (0.0)	0 (0.0)
Immunosuppression	27 (0.4)	3 (0.1)	1 (0.7)	1 (0.8)
Metastatic cancer	6 (0.5)	1 (0.4)	0 (0.0)	0 (0.0)
				
Hospital stay prior to unit admission (days; median [IQR])	1 (0–3)	1 (0–3)	1 (0–2)	0 (0–2)
CPR prior to admission (n [%])^a^	308 (8.3)	45 (5.8)	20 (13.8)	12 (8.5)
Intubation status (*n *[%])^b^				
Definitely intubated	2774 (73.9)	605 (78.1)	104 (13.8)	103 (72.5)
Probably intubated	278 (7.4)	56 (7.2)	13 (8.8)	9 (6.3)
Not intubated	700 (18.7)	114 (14.7)	30 (20.4)	30 (21.2)
				
Physiology^b^				
Lowest pH (median [IQR])	7.26 (7.18–7.33)	7.26 (7.18–7.33)	7.25 (7.19–7.33)	7.24 (7.15–7.32)
PaCO_2 _(kPa; median [IQR])^c^	8.7 (6.9–10.7)	8.6 (6.9–10.6)	9.1 (7.2–11.7)	7.6 (6.4–10.4)
PaO_2_/FiO_2 _gradient (kPa; median [IQR])^d^	22.9 (17.2–29.6)	21.2 (15.3–27.8)	20.9 (17.2–26.2)	23.3 (15.9–29.3)
Lowest serum albumin (g/l; median [IQR])	28 (23–33)	26 (21–31)	28 (25–32)	30 (25–34)
APACHE II				
APS (mean [SD])	13.4 (5.6)	14.3 (5.5)	13.5 (5.1)	14.6 (6.2)
APS (median [IQR])	12 (10–16)	13 (10–17)	13 (10–17)	14 (10–18)
Score (mean [SD])	19.4 (6.4)	20.3 (6.3)	19.9 (5.8)	20.7 (6.8)
Score (median [IQR])	19 (15–23)	19 (16–23)	19 (16–24)	20 (16–24)
APACHE III				
APS (mean [SD])	52.9 (21.5)	55.9 (21.6)	53.4 (20.0)	56.8 (21.5)
APS (median [IQR])	50 (39–64)	52 (42–68)	53 (41–66)	55 (42–68)
Score (mean [SD])	65.9 (23.0)	69.3 (23.0)	66.6 (21.8)	71.6 (22.9)
Score (median [IQR])	63 (50–78)	66 (54–83)	66 (52–80)	69.5 (55–84)
Organ system failures (*n *[%])				
0	457 (12.2)	85 (11.0)	19 (12.9)	15 (10.6)
1 Respiratory	1767 (45.2)	362 (45.0)	56 (37.4)	55 (33.8)
1 Other	214 (4.4)	60 (9.3)	5 (4.1)	9 (8.5)
2	1036 (27.6)	215 (27.7)	50 (34.0)	50 (35.2)
3 or more	309 (8.2)	54 (7.0)	17 (11.6)	17 (12.0)

^a^During 24 hours before unit admission. ^b^During first 24 hours following unit admission. ^c^Taken from ABG with lowest pH. ^d ^Taken from ABG with lowest PaO_2_. ABG, arterial blood gas; APACHE, Acute Physiology and Chronic Health Evaluation; APS, Acute Physiology Score; CPR, cardiopulmonary resuscitation; FiO_2_, fractional inspired oxygen; IQR, interquartile range; PaO_2_, arterial carbon dioxide tension; PaO_2_, arterial oxygen tension; SD, standard deviation; VF, ventricular failure.

### Outcome and activity

Overall, 827 (23.1%) patients died in the admitting CMP unit and 1322 (38.3%) died during the hospital admission (Table 2). The median (IQR) length of stay in the CMP unit was 4.0 (1.6–9.4) days and in hospital it was 16 (9–29) days. Readmissions within the same hospital stay accounted for 4.5% of all admissions. A decision was made to withdraw all active treatment in 422 (11.3%) patients, with the withdrawal decision being made a median (IQR) of 3.7 (1.2–7.9) days after admission to the CMP unit. The 2765 patients who were definitely intubated in the first 24 hours in the CMP unit had a median (IQR) stay in the CMP unit of 5.7 (2.5–11.4) days, as compared with 1.3 (0.6–2.6) days for the 697 patients who were definitely not intubated in the first 24 hours (Table 3).

**Table 2:** Outcome and activity for admissions with chronic obstructive pulmonary disease by subgroup

	All (*n *= 3752)	I. Pneumonia (*n *= 775)	II. Right VF (*n *= 147)	III. Left VF (*n *= 142)
Outcome				
Mortality in the CMP unit (*n *[%]) [95% CI]	827 (23.1) [21.7–24.5]	183 (24.5) [21.5–27.8]	50 (34.7) [27.0–43.1]	33 (24.1) [17.2–32.1]
Mortality in any hospital (*n *[%]) [95% CI]^a^	1322 (38.3) [36.7–40.0]	288 (40.1) [36.5–43.8]	73 (51.4) [42.9–59.9]	47 (36.4) [28.1–45.4]
Activity				
Length of stay				
In CMP unit (days; median [IQR])	4.0 (1.6–9.4)	5.5 (2.2–11.8)	4.1 (1.4–9.1)	3.3 (1.2–7.3)
Any hospital (days; median [IQR])^a^	16 (9–29)	19 (10–33)	13 (6–22)	14 (8–27)
Readmission within same hospital stay (*n *[%])	169 (4.5)	29 (3.7)	3 (2.0)	5 (3.5)
Transferred in (*n *[%])^b^	529 (14.1)	144 (18.6)	10 (6.9)	19 (13.5)
Transferred out (*n *[%])^b,c^	537 (18.6)	133 (22.7)	11 (11.3)	18 (17.0)
Decision made to withdraw all active treatment (*n *[%])	422 (11.3)	88 (11.4)	31 (21.1)	16 (11.3)
Time from unit admission to treatment withdrawal (days; median [IQR])^d^	3.7 (1.2–7.9)	5.0 (1.8–9.2)	4.0 (1.0–9.0)	3.0 (1.1–7.6)
Time from treatment withdrawal to death(hours; median [IQR])^e^	3.3 (1.1–8.9)	3.3 (1.0–9.4)	4.3 (1.5–7.6)	3.9 (2.0–6.8)

^a^Excluding 169 (4.5%) admissions who were readmissions to the intensive care unit (ICU) within the same hospital stay. ^b^From/to another critical care unit. ^c^Percentage of patients who were discharged from the unit alive. ^d^Calculated on all admissions with a decision to withdraw active treatment. ^e^Calculated on all admissions with a decision to withdraw active treatment, excluding 32 (7.6%) who were discharged from the unit alive. CI, confidence interval; CMP, Case Mix Programme; IQR, interquartile range; VF, ventricular failure.

**Table 3:** Length of stay in the Case Mix Programme unit by patient factors

	*n*	Length of stay (days; median [IQR])
Age>		
<55	484	3.6 (1.6–8.9)
55–60	410	4.7 (1.9–9.6)
60–64	574	4.1 (1.7–9.5)
65–69	761	4.5 (1.9–9.6)
70–74	785	4.2 (1.6–10.7)
75–79	557	3.0 (1.0–7.2)
80+	167	3.1 (1.3–7.2)
CPR before admission^a^		
No	3411	4.1 (1.6–9.5)
Yes	307	3.5 (1.1–8.8)
Intubation status^b^		
Not intubated	697	1.3 (0.6–2.6)
Probably intubated	276	2.0 (0.8–5.4)
Definitely intubated	2765	5.7 (2.5–11.4)
Organ system failures		
None	454	2.5 (1.0–7.6)
1 Respiratory	1688	4.0 (1.9–9.4)
1 Other	251	3.3 (1.0–8.6)
2	1036	5.1 (2.0–10.6)
3+	309	2.2 (0.8–7.5)

^a^During 24 hours prior to unit admission. ^b^During first 24 hours following unit admission. CPR, cardiopulmonary resuscitation; IQR, interquartile range.

### Risk factors for hospital mortality

Patients without any organ failure had a hospital mortality (95% confidence interval [CI]) of 33.8% (29.4–38.4%) whereas patients with single organ respiratory failure had a hospital mortality of 29.8% (27.6–32.1%; Table 4). As the number of organ failures increased the hospital mortality increased, with patients who had respiratory failure and three additional organ failures having a hospital mortality of 83.3% (65.3–94.4%). Eleven characteristics were found to have a significant independent association with increased hospital mortality in the multivariate analysis (Table 5): increased age; presence of severe respiratory disease; increased length of stay in hospital before admission to the CMP unit; cardiopulmonary resuscitation within 24 hours before admission; intubation status in the first 24 hours in the CMP unit; low pH; low PaO_2_/FiO_2 _gradient; low serum albumin; cardiovascular organ failure; neurological organ failure; and renal organ failure. Respiratory organ failure had a significant independent association with decreased hospital mortality (Table 5).

**Table 4:** Ultimate hospital mortality by organ system failures

Organ system failures	*n*	Mortality	(95% CI)
None	441	33.8	(29.4–38.4)
1 Respiratory	1632	29.8	(27.6–32.1)
1 Other	244	45.9	(39.5–52.4)
2	991	43.8	(40.7–46.9)
3+	303	70.0	(64.5–75.1)

CI, confidence interval.

**Table 5:** Multiple logistic regression model of patient factors in relation to ultimate hospital mortality

	Deaths	*n*	(%)	Unadjusted OR (95% CI)	Adjusted OR (95% CI)
Age				1.59 (1.46 – 1.73)	1.57 (1.44 – 1.73)
<55	103	469	(22.0)	per 10 year increase	per 10 year increase
55 – 60	108	395	(27.3)		
60 – 64	187	556	(33.6)		
65 – 69	281	745	(37.7)		
70 – 74	325	747	(43.5)		
75 – 79	293	537	(54.6)		
80+	96	162	(59.3)		
Sex					
Female	630	1742	(36.2)	Ref.	Ref
Male	763	1869	(40.8)	1.19 (1.03 – 1.39)	1.04 (0.88 – 1.22)
Severe respiratory disease					
No	827	2204	(37.5)	Ref.	Ref.
Yes	487	1200	(40.6)	1.15 (0.98 – 1.34)	1.23 (1.03 – 1.46)
Home ventilation					
No	1288	3337	(38.6)	Ref.	Ref.
Yes	26	67	(38.8)	1.05 (0.62 – 1.78)	1.16 (0.65 – 2.07)
Steroid treatment in previous 6 months					
No	1204	3110	(38.7)	Ref.	Ref.
Yes	110	294	(37.4)	0.96 (0.74 – 1.24)	1.00 (0.75 – 1.33)
Comorbidity: liver disease					
No	1309	3398	(38.5)	Ref.	Ref.
Yes	5	6	(83.3)	8.22 (0.96 – 70.47)	3.70 (0.39 – 35.37)
Comorbidity: cardiovascular disease					
No	1282	3326	(38.5)	Ref.	Ref.
Yes	32	78	(41.0)	1.03 (0.63 – 1.68)	0.88 (0.51 – 1.50)
Comorbidity: chronic renal failure					
No	1306	3393	(38.5)	Ref.	Ref.
Yes	8	11	(72.7)	3.29 (0.82 – 13.17)	2.21 (0.50 – 9.77)
Comorbidity: immunosuppression					
No	1299	3377	(38.5)	Ref.	Ref.
Yes	15	27	(55.6)	1.92 (0.89 – 4.17)	1.75 (0.71 – 4.28)
Comorbidity: metastatic cancer					
No	1310	3398	(38.6)	Ref.	Ref.
Yes	4	6	(66.7)	3.28 (0.60 – 17.96)	2.50 (0.43 – 14.66)
Length of stay before unit admission (days)				1.02 (1.01 – 1.03)	1.02 (1.01 – 1.03)
0	567	1618	(35.0)	per 1 day increase	per 1 day increase
1	256	753	(34.0)		
2	111	282	(39.4)		
3+	449	927	(48.4)		
CPR within 24 hours before admission					
No	1207	3291	(36.7)	Ref.	Ref.
Yes	176	301	(58.5)	2.38 (1.83 – 3.08)	1.83 (1.37 – 2.45)
Intubation status^a^					
Not intubated	206	684	(30.1)	Ref.	Ref.
Definitely/probably intubated	1187	2927	(40.6)	1.71 (1.38 – 2.13)	1.36 (1.07 – 1.73)
Lowest pH^a^				1.31 (1.23 – 1.40)	1.23 (1.13 – 1.35)
<7.18	391	758	(51.6)	per 0.1 decrease	per 0.1 decrease
7.18–7.25	294	797	(36.9)		
7.26–7.32	259	815	(31.8)		
= 7.33	297	877	(33.9)		

					

PaCO_2 _from ABG with lowest pH (kPa)^a^				1.09 (0.91 – 1.29)	1.01 (0.79 – 1.29)
<6.9	334	784	(42.6)	per 10 kPa increase	per 10 kPa increase
6.9–8.6	316	836	(37.8)		
8.7–10.6	270	783	(34.5)		
= 10.7	320	843	(38.0)		

PaO_2_/FiO_2 _gradient (kPa)^a^				1.17 (1.09 – 1.26)	1.13 (1.04 – 1.22)
<17.2	425	871	(48.8)	per 10 kPa decrease	per 10 kPa decrease
17.2–22.8	300	860	(34.9)		
22.9–29.5	293	856	(34.2)		
= 29.6	294	852	(34.5)		

Lowest albumin (g/l)^a^					
<20	178	303	(58.8)	2.73 (2.11 – 3.54)	1.94 (1.46 – 2.58)
20–24	208	477	(43.6)	1.49 (1.20 – 1.84)	1.28 (1.01 – 1.61)
= 25 or not recorded	1007	2831	(35.6)	Ref.	Ref.

Organ system failure: respiratory					
No	335	786	(42.6)	Ref.	Ref.
Yes	1058	2825	(37.5)	1.09 (0.87 – 1.36)	0.71 (0.55 – 0.91)

Organ system failure: cardiovascular					
No	881	2594	(34.0)	Ref.	Ref.
Yes	512	1017	(50.3)	2.21 (1.87 – 2.62)	1.48 (1.22 – 1.79)

Organ system failure: neurological					
No	1066	2978	(35.8)	Ref.	Ref.
Yes	327	633	(51.7)	1.85 (1.53 – 2.24)	1.36 (1.10 – 1.68)

Organ system failure: renal					
No	1190	3346	(35.6)	Ref.	Ref.
Yes	203	265	(76.6)	5.17 (3.70 – 7.23)	3.83 (2.67 – 5.47)

Organ system failure: haematological					
No	1362	3550	(38.4)	Ref.	Ref.
Yes	31	61	(50.8)	1.65 (0.94 – 2.89)	1.30 (0.69 – 2.45)

Ultimate hospital mortality excludes admissions who were readmissions to the intesnive care unit within the same hospital stay, admissions whose ultimate hospital discharge status was missing, and admissions for whom any of the entered risk factors were missing. ^a^During first 24 hours following unit admission. ABG, arterial blood gas; CI, confidence interval; CPR, cardiopulmonary resuscitation; FiO_2_, fractional inspired oxygen; OR, odds ratio; PaCO_2_, arterial carbon dioxide tension; PaO_2_, arterial oxygen tension.

Case mix adjusted comparison of the three subgroups pneumonia, right ventricular failure and left ventricular failure gave adjusted odds ratios for ultimate hospital mortality (95% CI) of 1.04 (0.86–1.27), 1.59 (1.06–2.38) and 0.77 (0.50–1.18), respectively. This showed patients with right ventricular failure to have a significantly increased risk for hospital mortality after case mix adjustment.

## Discussion

This study describes the outcomes of 3752 COPD patients admitted to UK ICUs over a 5-year period and includes analyses that describe the association between patient characteristics available within the first 24 hours of ICU admission and hospital outcome.

Overall, patients with COPD admitted to the 128 ICUs in this study had a 77% chance of surviving to leave ICU and a 61% chance of surviving to leave hospital. Comparisons of ICU outcomes between studies are difficult to interpret because ICU beds are used in different ways in different countries and subtle differences in inclusion criteria can make populations differ in prognostically important ways. The SUPPORT (Study to Understand Prognoses and Preferences for Outcomes and Risks of Treatment) study [5] recruited consecutive hypercapnic COPD patients admitted to five US hospitals. Of the 1016 hospitalized hypercapnic COPD patients, 526 (51.8%) were admitted to ICU and 348 (66.2%) of those admitted to the ICU were intubated. This intubation rate contrasts with 81.1% being probably or definitely intubated in the first 24 hours in the present study. Caution must also be exercised in comparisons focusing on the apparently more homogeneous intubated groups. For example, although the study conducted by Seneff and coworkers [7] included patients with COPD exacerbations, patients who had right ventricular failure or pneumonia were excluded. In our study, patients with right ventricular failure had a significant, independent increased risk for death, and the exclusion of these patients may go some way to explaining the difference in hospital mortality in intubated patients between the Seneff study (31.8%, 95% CI 24.8–39.3%) and this study (40.6%, 95% CI 38.8–42.3%). Although almost all studies report hospital mortality, measures such as 30- or 60-day mortality are more robust for comparison purposes because the definition of hospital mortality may be influenced by local discharge practices. For example, Nevins and Epstein [6] reported a comparatively low hospital mortality of 27.7% (95% CI 21.1–35.2%) in 166 intubated COPD patients from one US centre, but 38% of patients were transferred to chronic care facilities. In contrast, Ely and coworkers [15] reported a hospital mortality of 38.6% (95% CI 24.4–54.5%) for 44 intubated COPD patients from a US academic centre, which is similar to that observed in the present study. Esteban and coworkers [16] studied 522 patients with COPD receiving mechanical ventilation in 361 ICUs in 20 countries and found an overall hospital mortality of 28% (95% CI 24–32%) with a median (IQR) Simplified Acute Physiology Score II of 38 (31–49), as compared with 42 (35–52) for the intubated patients in the present study.

Differences in length of stay between studies are likely to be influenced by the case mix and supply side factors discussed above. The 522 mechanically ventilated COPD patients from the study by Esteban and coworkers [16] had a median (IQR) length of ICU stay of 8 (5–13) days, as compared with 4.0 (1.6–9.4) days observed in our study. However, the total hospital length of stay was similar in both studies, with the Esteban study having a median (IQR) hospital stay of 17 (10–27) days versus 16 (9–29) days in the present study. Longer ICU stay (median 7 days, IQR 4–14) and similar hospital stay (median 14 days, IQR 9–29) are also apparent in the study by Nevins and Epstein [6]. The greater proportion of the total hospital stay spent in European and North American ICUs compared with the UK may well reflect the lower proportion of all hospital beds designated for critical care in the UK.

This study demonstrates that age has an independent relationship with hospital death. This increased risk is similar to that observed in the SUPPORT study, which calculated the independent relative hazard of death to be 1.22 (95% CI 1.05–1.41) per 10-year increase [5]. A number of studies of COPD outcome [6,8,9,17] did not find a relationship between age and hospital mortality. There is potential for prognostic studies to produce conflicting results because of inaccuracy in the measurement of patient characteristics, inadequate measurement of and adjustment for confounding, and lack of power [18,19]. Most commentators suggest that a minimum of between 15 and 20 deaths will be required per risk factor analyzed [20,21]. In three of the studies that did not find age to predict mortality [6,8,9], age was analyzed as a univariate predictor with between 20 and 46 deaths. In the study by Breen and coworkers [17], in which age was analyzed in a multivariate logistic regression, there were only 15 hospital deaths.

Of chronic diseases existing before hospitalization, only severe respiratory disease had a significant independent relationship with hospital death. Patients with severe respiratory disease have shortness of breath performing most ADL, and the increased risk for mortality for patients with severe respiratory disease is consistent with the increased hazard of death associated with impairment in ADL identified in SUPPORT (relative hazard per additional ADL impairment: 1.14, 95% CI 1.03–1.26) [5]. Prior cardiovascular disease was only present in 78 patients (2% of the total) and did not have a significant independent association with outcome. Although a 2% prevalence of cardiovascular comorbidity may seem surprisingly low in a COPD population, it is should be remembered that, to qualify for this comorbidity, patients had to have impairment equivalent to New York Heart Association functional class IV. A total of 294 patients (7.8%) had received 0.3 mg/kg steroid daily for the 6 months prior to admission. Oral steroids have been suggested to confer an increased risk for hospital mortality in a small study conducted in intubated COPD patients using univariate analysis [9]. It is possible that the lack of association of oral steroids with death in this study may represent a true absence of association. However, the use of oral steroids in a heterogeneous population, which might have included both COPD patients with a marked asthmatic component and a better than average prognosis, as well as very severe COPD patients with a worse than average prognosis, could lead to no net effect being detected, as of course could incomplete documentation of steroid use in the population as a whole.

In a study of 362 COPD admissions to US critical care units [22] the number of days in hospital before critical care admission was reported to be significantly associated with hospital mortality, although the increased risk per day was not reported. This study confirms an association between days in hospital before ICU and hospital mortality, but it shows the independent risk to be relatively small with patients having only a 2% (95% CI 1–3%) increased odds of hospital death for each day in hospital before ICU admission.

Brochard and coworkers [23] suggested that invasive ventilation was associated with increased mortality when compared with noninvasive ventilation, but there were differences in severity between groups. Seneff and coworkers [7] found that intubation on day 1 was not an independent predictor of hospital mortality (*P *= 0.07) in a multiple regression model, with 170 patients intubated on day 1 and 54 deaths among intubated patients. Afessa and coworkers [8] also failed to find an independent association between intubation and death in a study of 250 episodes of respiratory failure in 180 COPD patients, in which 153 patients episodes involved intubation and 31 patients died. In this study, 3052 patients were definitely or probably intubated on day 1 and intubation on day 1 was associated with an independent odds ratio (95% CI) of 1.36 (1.07–1.73) for hospital mortality. Given that the risk for ventilator-associated pneumonia has been estimated to be 3% per day [24], it would not be surprising to find increased hospital mortality associated with intubation, and it is possible that the Seneff and Afessa studies were underpowered to detect this effect.

Cardiovascular, neurological and renal organ failures were all associated with a significant, independent increased risk for hospital mortality. Treatment withdrawal was reported to occur in 11.3% of admissions, and if the development of organ failure were to precipitously trigger withdrawal decisions then interpretation of the prognosis associated with organ failure would be difficult. Treatment withdrawal was most common in patients with acute renal failure, but treatment withdrawal accounted for fewer than one-third of the deaths, and recalculation of the risk for death after removing all patients with withdrawal decisions resulted in little change in the OR for death associated with acute renal failure. Respiratory organ failure had a significant independent association with lower hospital mortality, with patients with isolated respiratory failure having a hospital mortality of 29.8%, as compared with 33.8% for patients without any organ failures. It is possible that patients admitted to ICU with COPD but without respiratory organ failure had more severe illness in other organ systems. For example, although the regression model adjusts for the presence or absence of acute renal failure, the severity of renal failure may still constitute residual confounding. Afessa and coworkers [8] also used the Knaus organ failure classification [14] and found an independent increased odds of hospital death of 5.5 per organ failure (excluding respiratory organ failure). Seneff and coworkers [7] found that the development of nonrespiratory organ dysfunction was the major predictor of hospital mortality in 362 COPD admissions to US ICUs. This is also consistent with a study of patients with heterogeneous causes of respiratory failure [25], in which other organ failures were found to be more important than the severity of the respiratory failure.

Although practice may have changed over the period during which data were sampled, for example because increasing use of noninvasive ventilation, this analysis was not conceived to explore, detect and report secular trends. This may be a suitable topic for further research in this area.

When patients with obstructive lung disease present with decompensated type II respiratory failure, it can be very difficult to distinguish those with 'pure' COPD (without any significant reversibility) from those with a mixture of COPD and asthma (patients with a major component of fixed obstruction that coexists with some reversibility). It is likely that patients with greater reversibility will tend to be younger, possibly respond better to steroids and have a better overall prognosis. Unfortunately, the CMP data do not allow this distinction to be explored, and there is no real agreement about how this distinction should be made even when a patient has had full lung function – a distinction that is even harder in the acute setting when information regarding lung function is rarely available. Additional prospective research may be required to further illuminate this problem.

## Conclusion

This study demonstrates that patients admitted to ICU with COPD and single organ respiratory failure have a 70% chance of leaving hospital alive and a median length of stay in hospital of 16 days. Although patients entering critical care will represent a selected group of all those patients admitted to hospital with respiratory failure, it is reassuring that the independent risk factors for mortality have much in common with those identified in the SUPPORT cohort, which included all COPD admissions with hypercapnic respiratory failure irrespective of whether the patient was admitted to ICU.

## Key messages

• Non-surgical COPD patients account for 2.9% of all ICU admissions in the UK

• Intubated patients have a median stay in intensive care of 6 days

• COPD patients admitted to ICU have a mortality of 23% in the admitting ICU and 38% in hospital, which is similar to the mortality of patients with all diagnoses admitted to ICU

## Abbreviations

ADL = activities of daily living; APACHE = Acute Physiology and Chronic Health Evaluation; CI = confidence interval; CMP = Case Mix Programme; CMPD = Case Mix Programme Database; COPD = chronic obstructive pulmonary disease; FiO_2 _= fractional inspired oxygen; ICU = intensive care unit; IQR = interquartile range; PaO_2 _= arterial oxygen tension.

## Competing interests

The author(s) declare that they have no competing interests.

## Authors' contributions

MW drafted the manuscript. DH performed the analyses and contributed to drafting the manuscript. All authors participated in the design and interpretation of the study, and critical revision of the manuscript, and read and approved the final manuscript.
